# Genomic perspectives on the global dissemination of *Elizabethkingia anophelis*: unveiling inherent multidrug resistance and virulence determinants

**DOI:** 10.1186/s12866-026-04846-7

**Published:** 2026-02-27

**Authors:** Shaohua Hu, Xiaohua Meng, Hao Xu, Shujun Ni, Yonghong Xiao, Beiwen Zheng

**Affiliations:** 1 State Key Laboratory for Diagnosis and Treatment of Infectious Diseases, National Clinical Research Center for Infectious Diseases, Collaborative Innovation Center for Diagnosis and Treatment of Infectious Diseases, the First Affiliated Hospital, Zhejiang University School of Medicine, Hangzhou, China; 2 Yuhang Institute of Medical Science Innovation and Transformation, Hangzhou, China; 3 Department of Structure and Morphology, Jinan Microecological Biomedicine Shandong Laboratory, Jinan, China; 4 Research Units of Infectious Diseases and Microecology, Chinese Academy of Medical Sciences, Beijing, China

**Keywords:** *Elizabethkingia anophelis*, Resistance genotypes, Virulence genes, Phenotypes-genotypes interaction

## Abstract

**Background:**

*Elizabethkingia anophelis* has emerged as a formidable pathogen responsible for severe, life-threatening infections in immunocompromised populations. However, the genetic underpinnings of its virulence and antimicrobial resistance remain poorly characterized. Leveraging our previously assembled collection of 197 *E. anophelis* isolates with complete genome sequences, we performed an exhaustive, large-scale comparative analysis across global datasets to systematically map resistance determinants and virulence factors. Additionally, we constructed an integrated coexpression network to elucidate genotype-phenotype correlations in antibiotic resistance patterns.

**Results:**

The distribution of virulence-associated genes displays moderate phylogenetic specificity, exhibiting constrained variation within established primary clades. Importantly, virulence gene profiles demonstrate little discernible association with specimen source, sample category, or geographic origin. Although diverse antimicrobial resistance genotypes were detected, these exhibited negligible lineage specificity with minimal differentiation among core phylogenetic clusters. Coexpression network analysis suggests that *E. anophelis* resistance to cephalosporins, carbapenems, and aztreonam primarily arises from the constitutive expression of chromosomally encoded resistance determinants. In contrast, these chromosomally encoded resistance mechanisms appear to exert either negligible or clinically insignificant effects on susceptibility patterns for the remaining fourteen tested antimicrobial agents.

**Conclusions:**

This study demonstrates that the multidrug resistance and pathogenic potential of *E. anophelis* are predominantly intrinsic traits, regulated by complex, multilayered biological mechanisms. Comprehensive elucidation of the bacterium’s virulence and resistance pathways necessitates the discovery of novel molecular targets followed by systematic, in-depth characterization.

**Supplementary Information:**

The online version contains supplementary material available at 10.1186/s12866-026-04846-7.

## Introduction

The *Elizabethkingia* genus is a group of Gram-negative bacilli commonly found in water and soil, characterized by their aerobic, oxidase-positive, non-fermenting, and non-motile nature [1]. At present, the *Elizabethkingia* genus is mainly comprised six species, including: *Elizabethkingia anophelis* (*E. anophelis*), *Elizabethkingia meningoseptica*, *Elizabethkingia miricola*, *Elizabethkingia occulta*, *Elizabethkingia ursingii*, and *Elizabethkingia bruuniana* [2, 3]. Although constituting rare pathogens, the newly identified species *E. anophelis* has emerged as a significant focus in recent years. *E. anophelis* can cause life-threatening infections and have been implicated in hospital-acquired infection since their description by Thierry et al. in 2013 when they were first reported as a cause of neonatal meningitis in the Central African Republic [4]. This organism has been associated with a spectrum of nosocomial infections in immunocompromised patients, encompassing neonatal meningitis, bacteremia, hospital-acquired pneumonia, urinary tract infections, sepsis, and systemic infections [2, 4–9]. Notably, it has been linked to documented nosocomial outbreaks across multiple regions, particularly in the United States (Wisconsin) [10], Singapore [11], South Korea [8], Taiwan [12], and France [13].

As an understudied pathogen, *E. anophelis* has recently garnered increasing attention due to rising infectious reports. This microorganism exhibits resistance to a broad spectrum of antibiotics, such as cephalosporins, carbapenems, fluoroquinolones, and aminoglycosides [14–17]. Disturbingly, the mortality rate linked to *E. anophelis* infections has exhibited a concerning upward trend in recent years. During the 2015–2016 U.S. outbreak, the case fatality rate stood at 31% (20/65) [18], while a 2022 outbreak in France recorded an even more alarming rate of 45% (9/20) [13]. Notably, this pathogen has been isolated from hospital tap water and contaminated swab kits, suggesting these may serve as critical reservoirs for nosocomial transmission [12, 19]. The convergence of pervasive antimicrobial resistance and high lethality poses formidable challenges for both clinical management and infection control. Yet, research remains scarce on the antibiotic susceptibility profiles, resistance determinants, and virulence mechanisms of *E. anophelis*. The molecular basis of its multidrug resistance and pathogenic potential remains elusive, underscoring an urgent need for expanded investigation into this emerging threat.

In prior research, we systematically explored the interplay between antimicrobial resistance (AMR) and biofilm formation capacity across an extensive cohort of *E. anophelis* isolates derived from Chinese hospitalized patients [20]. The study encompassed phenotypic susceptibility profiling of 197 clinical isolates (2010–2019) against 19 clinically relevant antibiotics, complemented by whole-genome sequencing and integration of publicly available genomic datasets to facilitate global population genomics and transmission dynamics analysis [21]. This subsequent investigation expands upon foundational work through a multinational genomic epidemiology study, elucidating phenotype-genotype concordance in drug resistance alongside virulence gene architecture—an endeavor critical for deciphering the organism’s mechanistic underpinnings of pathogenicity and resistance. Notably, this constitutes the inaugural global mapping of *E. anophelis* resistance determinants and virulence factors, contextualized within phylogenetic evolution, bio-geographical distribution, and clinical specimen typology. Furthermore, we pioneer a novel coexpression network framework correlating genotypic resistance markers with phenotypic susceptibility profiles at an unparalleled population scale. Our findings indicate that multidrug resistance and pathogenicity largely conserved across lineages.

## Materials and methods

### Isolates and genomes

This study analyzed a global collection of 318 *E. anophelis* strains, comprising 197 previously sequenced clinical isolates from our prior research and 121 publicly available genomes from NCBI [21]. The isolates originated from four continents across more than ten countries, with the study workflow illustrated in Fig. S1. The dataset included 197 clinical *E. anophelis* isolates collected in Zhejiang, China between 2010 and 2019, all subjected to whole-genome sequencing. Additionally, we retrieved all accessible bacterial genomes that could be obtained at the time, consisting of 65 assembled genomes from GenBank and 56 raw sequencing datasets from SRA. Comprehensive sample metadata regarding geographic origins and clinical sources are documented in our previous publication [21]. The antimicrobial susceptibility of the 197 clinical isolates was determined using the standard agar dilution method, with testing conducted for nineteen common antibiotics [20].

### Resistance genes and virulence genes identification

The in silico detection of AMR genes was conducted by analyzing genome sequences against the Comprehensive Antibiotic Resistance Database (CARD) using Resistance Gene Identifier (RGI) software version 5.1.0 [22, 23]. Protein sequence alignments were performed using BLAST (https://blast.ncbi.nlm.nih.gov/Blast.cgi). All analyses employed default parameters in the RGI portal, with the following screening criteria: e-value < 1e-30, protein identity > 50%, query coverage > 50%, subject coverage > 50%, match length > 100 amino acids, and identical residues > 100 amino acids. The reproducible workflow for resistance gene analysis and command-line RGI implementation are available at https://github.com/arpcard/rgi. To evaluate AMR-associated point mutations in core genes, we BLAST these target genes against the NCBI protein database for *E. anophelis* to identify mutations. Virulence-associated genes were predicted using BLASTp against the virulence factor database (VFDB) Protein Set B dataset [24, 25], with stringent filtering thresholds matching those applied for AMR gene detection (all parameters > 50%, length > 100 amino acids). Comparative analysis of resistance genes and virulence factors among *E. anophelis* strains was performed using R statistical software. And the workflow for the virulence genes prediction can be found at the following link: https://github.com/haruosuz/vfdb.

### Phylogenomic reconstruction and network analysis

Phylogenetic reconstruction was conducted using maximum-likelihood analysis of single nucleotide polymorphisms (SNPs) with PhyML [21] under the HKY85 substitution model. The workflow involved read mapping, SNP calling, and stringent filtering of 318 *E. anophelis* isolates, followed by concatenation of high-confidence SNP alleles for tree construction. Paired-end reads were mapped to the reference genome of *E. anophelis* CSID_3015183678 using MUMmer [26] (version 3.23) for SNP analysis. Sites differing between sample sequences and the reference were identified and preliminarily filtered to detect candidate SNP loci. Flanking 100 bp sequences on either side of each reference SNP site were extracted and aligned to the assembled results using BLAST for validation. Read mapping, SNP calling, and preliminary filtering were conducted using the RedDog phylogenomics pipeline (https://github.com/katholt/RedDog). The resulting phylogeny elucidated species divergence patterns through differential base‑substitution rates, clarifying evolutionary relationships and topological structures. Finally, resistance and virulence gene distributions were visualized as thermographic heatmaps using R software (v3.5.3) with the heatmap package.

The genotype-phenotype co-expression network was constructed through systematic pairwise comparisons and statistical analyses of strain-specific genomic profiles and antibiotic resistance patterns. Strains were classified into four distinct clusters based on the congruence levels between genotypic markers and phenotypic resistance profiles, each demonstrating unique genotype-phenotype interaction patterns in antimicrobial resistance. For *E. anophelis*, the antibiotic phenotype-genotype interaction (PGI) network was visualized using Cytoscape 3.8.0 (http://cytoscape.org) [27], implemented through the following analytical workflow: (i) Network construction integrating antibiotic phenotypes with genotypes, (ii) Visualization optimization using yFiles Organic Layout algorithm, (iii) i Incorporation of gene expression data and functional annotations, (iv) Advanced graphical customization of topological features (node shapes, color schemes, edge styles, and quantitative attributes). The computational pipeline and visualization scripts are publicly accessible at https://github.com/Cytoscape. Complementary flow analysis was performed through Sankey diagram generation using R (v3.5.3) with ggalluvial and networkD3 packages for multidimensional data representation.

## Results

### Genetic determinants of antimicrobial resistance

**Fig. 1:**
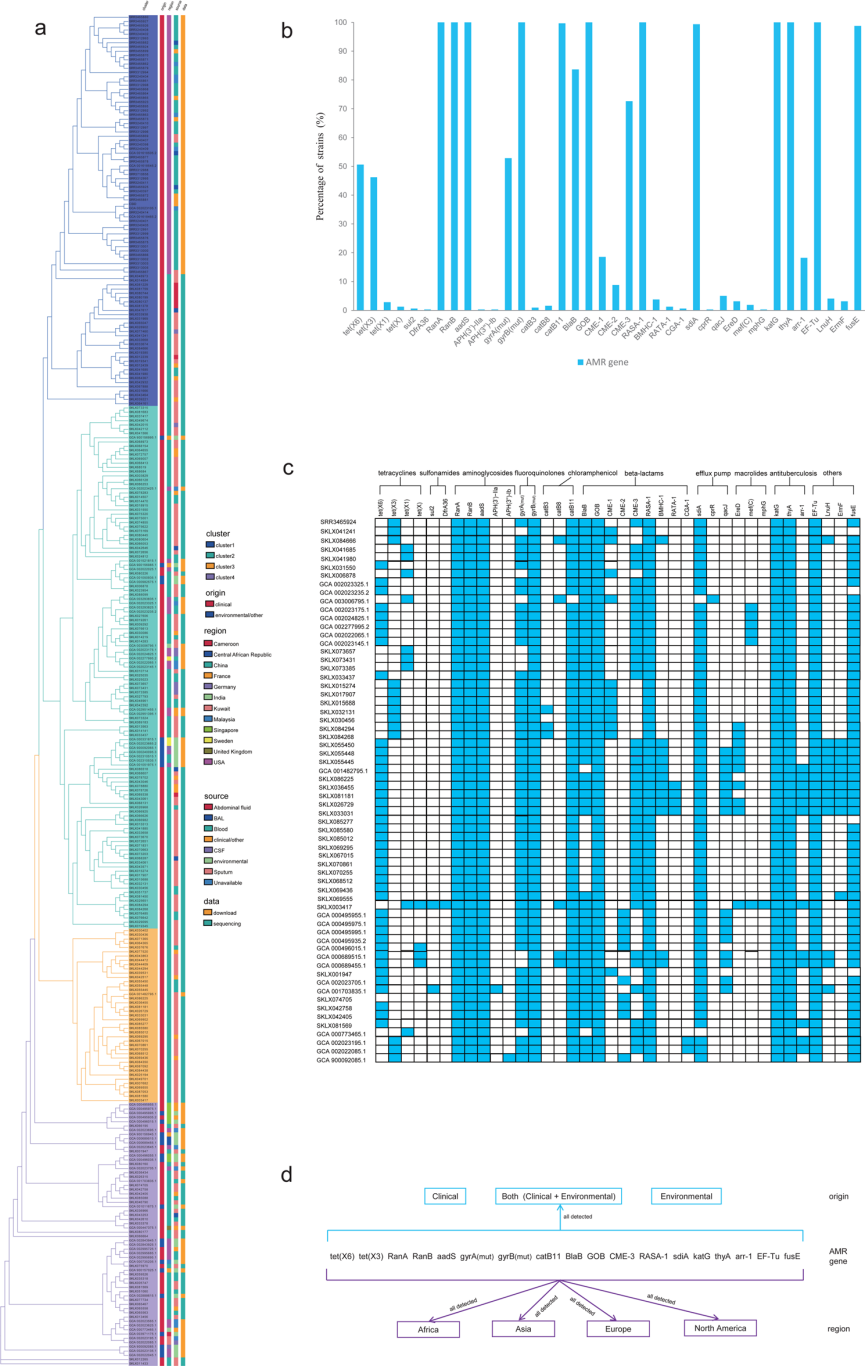
Phylogenetic distribution of antimicrobial resistance (AMR) genes in global *E. anophelis*. **a** Maximum-likelihood SNP phylogeny of 318 *E. anophelis* isolates. Column colors correspond to legend annotations at right. **b** Prevalence of AMR-associated genes among global *E. anophelis* strains. The x-axis denotes AMR gene categories; the y-axis indicates the proportion of strains harboring each gene; *gyrA* (mut): *gyrA* mutants, *gyrB* (mut): *gyrB* mutants. **c** Distribution of resistance genes in strains containing unique gene sets. Genes are indicated by blue (present) or white (absent) cells. Corresponding antibiotic classes are annotated above each gene: tetracyclines, sulfonamides, aminoglycosides, fluoroquinolones, chloramphenicol, β-lactams, efflux pumps, macrolides, antituberculosis agents, and others. **d** Sample sources versus resistance gene associations. Central genes represent commonly identified resistance determinants; top sections indicate strain origins, while bottom sections denote geographical sources

Genomic characterization of 318 globally distributed *E. anophelis* isolates identified a comprehensive array of AMR determinants, encompassing both horizontally transferred resistance genes and chromosomally encoded point mutations. The analysis uncovered substantial genotypic diversity in AMR markers, yet revealed striking conservation across phylogenetic lineages, with negligible variation observed among primary strain groupings (Fig. 1, S2). Phylogenetic reconstruction of 197 Chinese isolates demonstrated a panmictic population structure organized into four major clades containing phylogenetically diverse sub-clusters. Crucially, the evolutionary topology showed no significant association with geographical provenance, isolation source, or regional epidemiology (Fig. 1a). All clades maintained nearly identical AMR gene complements and resistance profiles, notwithstanding minor variations in individual resistance determinants. Spatial analysis of resistance gene distribution revealed no correlation with geographic origin, nor discernible differentiation between clinical and environmental isolates (Fig. 1d, S2). These collective findings strongly suggest that multidrug resistance mechanisms are largely conserved across *E. anophelis* lineages, independent of strain origin or ecological niche.

The study identified over thirty distinct AMR genes in *E. anophelis* isolates, with most genes detected in > 50% of samples. Notably, *RanA*, *RanB*, *catB11*, *bla*_*CME−3*_, and *tet(X6)* demonstrated high prevalence rates (100%, 100%, 99.7%, 72.6%, and 50.6%, respectively; Fig. 1b). All isolates harbored carbapenemase-encoding genes (*bla*_*BlaB*_, *bla*_*GOB*_), confirming resistance to both imipenem and meropenem. Over 99% of strains possessed intrinsic resistance genes against tetracyclines, chloramphenicol, aminoglycosides, β-lactams (penicillins/cephalosporins), and antituberculosis agents, with universal detection of *aadS*, *RASA-1*, *katG*, *thyA*, and *EF-T*u across all analyzed strains (Fig. 1b and c). Mutations in *gyrA* and *gyrB* contribute to fluoroquinolone resistance. Although *gyrB* mutations (V423F, I139R, V301L, V457L) were ubiquitous across all isolates, novel distinct mutations (D461H, D434H) occurred only in strain SKLX014884 (Table S1). Multiple *gyrA* mutations were identified in over half of the strains. Predominant mutations included S83I, S83R, D87N, T409A, N701S, V833L, I834L, A841V, and I842A, while G81D, Y100X, P101X, L102X, R121X, E282D, A709S, N800I, D829H, and P835Q were detected only in several isolates (Table S1). Rare resistance determinants—*APH(3’)-IIa*, *APH(3’’)-Ib* (aminoglycoside resistance), *DfrA3*6 (sulfonamide resistance), *cprR* (multidrug efflux), and *mphG* (macrolide resistance)—were each identified in single isolates, whereas *sul2* and *CGA-1* appeared in only two strains (Fig. 1c). Geographically, all rare variants except the *APH(3’’)-Ib-*positive isolate originated exclusively from Asian specimens.

### Antibiotic phenotypes and genotypes coexpression network

**Fig. 2:**
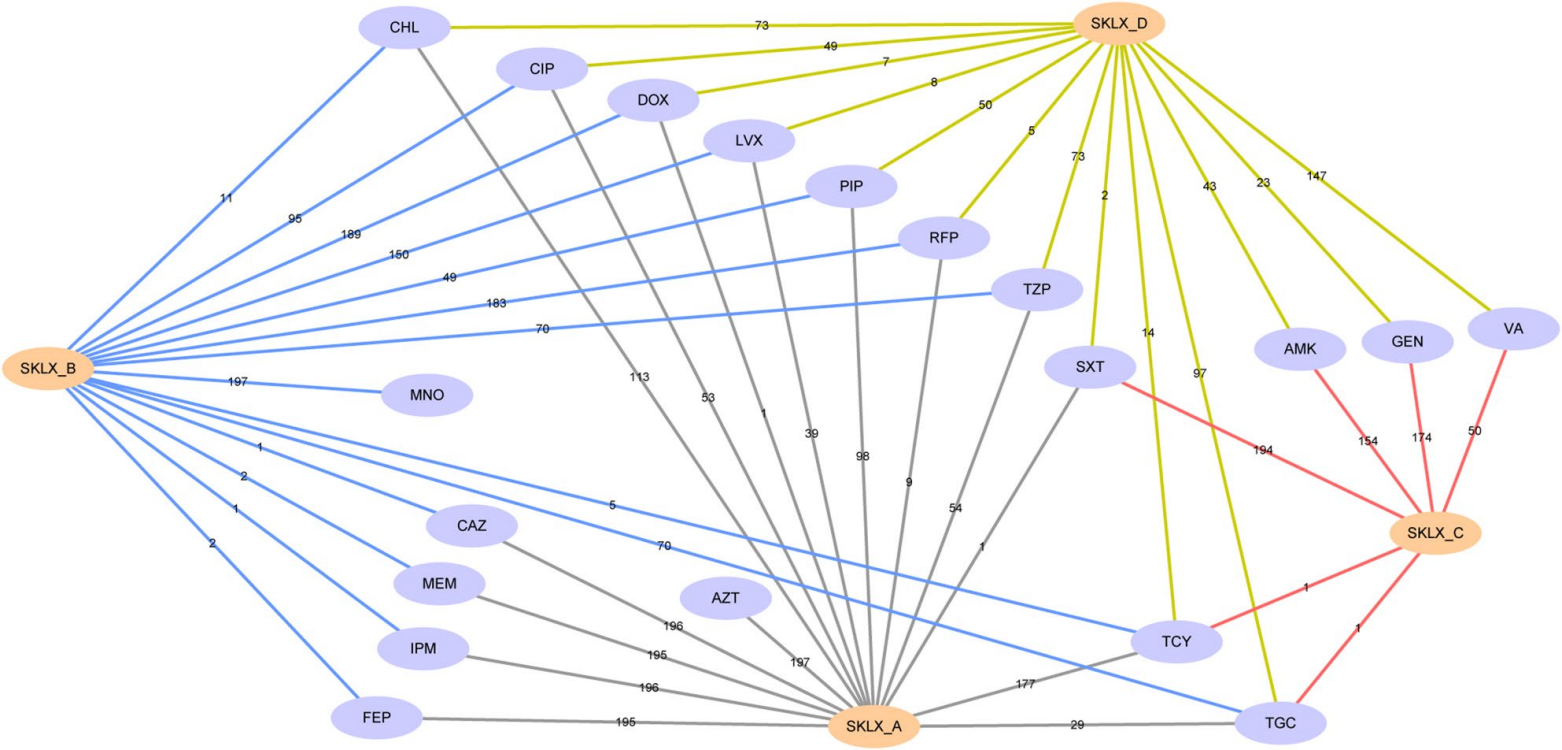
Coexpression network linking antibiotic phenotypes and genotypes in 197 clinical *E. anophelis* isolates (Cytoscape analysis). Orange circles (SKLX_A–D) classify isolates by resistance profiles: SKLX_A: Resistant strains with detected corresponding resistance genes. SKLX_B: Sensitive strains carrying resistance genes. SKLX_C: Resistant strains lacking detectable resistance genes. SKLX_D: Intermediate/full susceptibility strains without resistance genes, or intermediately susceptible strains harboring relevant resistance genes. Colored lines (A–D) indicate isolate counts per group. Nattier blue circles denote antibiotics: PIP (piperacillin), TZP (piperacillin-tazobactam), CAZ (ceftazidime), FEP (cefepime), IPM (imipenem), MEM (meropenem), AZT (aztreonam), GEN (gentamicin), AMK (amikacin), MNO (minocycline), DOX (doxycycline), TCY (tetracycline), TGC (tigecycline), CIP (ciprofloxacin), LVX (levofloxacin), SXT (trimethoprim-sulfamethoxazole), RFP (rifampin), VA (vancomycin), CHL (chloramphenicol)

To elucidate the antibiotic resistance mechanisms in *E. anophelis*, we established a coexpression network of shared AMR determinants using genotypic and phenotypic data from 197 collected strains (Fig. 2, S3). The network analysis was restricted to our isolated strains, as the remaining 121 NCBI-sourced strains lacked experimentally validated resistance measurements. Comprehensive antibiotic resistance profiles for each *E. anophelis* strain are presented in Fig. S4. Comparative analysis of AMR profiles and genomic resistance genes revealed that *E. anophelis* resistance appears predominantly intrinsically encoded rather than acquired. Resistance to cephalosporins, carbapenems, and aztreonam was strongly associated with chromosomally encoded resistance genes, as evidenced by the network (Fig. 2, S3). Specifically, over 95% of resistant strains harbored corresponding resistance determinants for ceftazidime, cefepime, imipenem, meropenem, and aztreonam. In contrast, resistance genes for piperacillin and piperacillin-tazobactam were detected in only 49.7% and 28.9% of resistant isolates, respectively. Notably, while aminoglycoside resistance genes were universally present across all isolates, phenotypic resistance to gentamicin and amikacin was observed in just 88.3% and 78.2% of strains, respectively (Fig. S4), suggesting additional regulatory or post-transcriptional mechanisms may influence resistance expression.

Fascinatingly, while tetracycline-associated resistance gene *tetX* was detected in nearly all 197 isolates, only 89.9% exhibited phenotypic resistance to tetracycline itself. For tigecycline, merely twenty-nine isolates demonstrated concordance between genotype and resistance profile (Fig. 2). Notably, these strains displayed remarkable sensitivity to tetracycline derivatives—100% susceptibility to minocycline and 95.9% to doxycycline (Fig. S4), suggesting that *E. anophelis* tetracycline resistance mechanisms may operate independently of conventional resistance determinants. Fluoroquinolone resistance (levofloxacin/ciprofloxacin) presented a similar paradox: despite near-universal carriage of resistance genes, only a minority of strains manifested corresponding phenotypic resistance. This genotype-phenotype discordance extended to other antibiotics—trimethoprim-sulfamethoxazole (98.5% resistant despite gene detection in just one isolate), rifampin (92.9% sensitive despite universal gene presence), and chloramphenicol (57.3% resistant despite universal gene detection) (Fig. S3). Collectively, among our 197 *E. anophelis* isolates, antibiotic resistance phenotypes rarely correlated with identified genetic determinants, implying complex, multifactorial resistance mechanisms beyond canonical gene-mediated pathways.

### Virulence genes and inherent pathogenicity

**Fig. 3:**
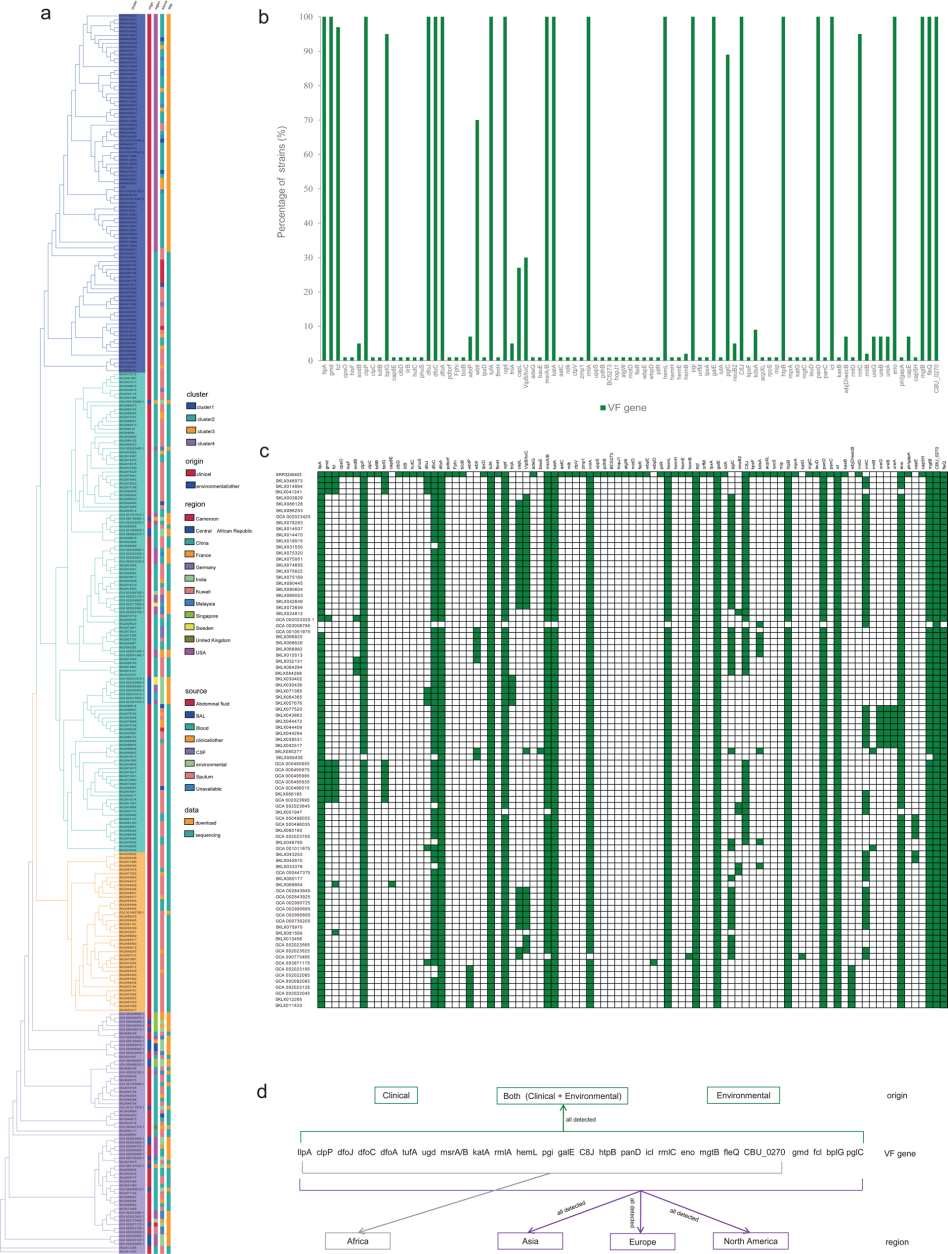
Phylogenetic distribution of virulence genes across 318 globally sampled *E. anophelis* isolates. **a** Maximum-likelihood SNP phylogeny of global *E. anophelis* isolates. Color-coded columns (denoting cluster, origin, region, source, and dataset descriptors) align with Fig. 1 conventions. **b** Prevalence of virulence-associated genes among 318 *E. anophelis* strains. The x-axis represents gene distribution, while the y-axis quantifies the proportion of strains possessing each gene. VF: Virulence factor. **c** Distribution of virulence-related genes in strains containing corresponding unique determinants. All identified genes are annotated as dark green (present) or white (absent). **d** Schematic illustrating associations between sample sources and virulence gene distribution. Central panel elements denote commonly detected virulence genes; upper and lower sections indicate isolate origins and strain source designations, respectively

A comprehensive mapping of virulence-associated genes was conducted across a global collection of *E. anophelis* isolates, revealing distinct lineage-specific patterns with minimal intra-cluster variation (Fig. 3, S5). For example, Cluster 1 (blue) demonstrated remarkable genetic homogeneity in virulence profiles, barring minor exceptions. Notably, virulence gene distribution exhibited no significant association with sample origin (clinical/environmental), type, or geographic region, implying intrinsic pathogenic potential in *E. anophelis* (Fig. 3d, S5). Of particular significance, U.S. outbreak strains harbored an elevated repertoire of documented virulence factors, potentially explaining their enhanced pathogenicity and transmission capacity. The genomic analysis identified 89 virulence-related genes across all 318 isolates, with *clpP*, *dfoA*, *tufA*, *katA*, *rmlA*, *hemL*, *pgi*, *galE*, *htpB*, *panD*, *icl*, and *fleQ* universally conserved. High-prevalence genes (*ilpA*, *msrA/B*, *eno*, *mgtB*) occurred in > 98% of strains, while rare determinants (*cpsO*, *hisF*, *kdtB*, *cap8E*, *lirB*, *flmH*, *bauE*, *wbpD*, *mprA*, *katG*, etc.) appeared singularly (Fig. 3b, c). Notably, a type IV secretion system (T4SS) effector, CBU_0270 *Coxiella* Dot/Icm type IVB secretion system translocated effector, was universally detected in *E. anophelis* isolates, suggesting a possible mechanism for enhanced protein secretion or conjugative transfer that could influence host-cell invasion efficiency. This restricted distribution contrasts with the widespread conservation of other virulence machinery, highlighting potential evolutionary specialization in pathogenic mechanisms.

Our findings revealed that *E. anophelis* harbors an array of virulence factors analogous to those documented in established bacterial pathogens. Functionally categorized, these virulence-associated genes predominantly encode mechanisms for capsule polysaccharide synthesis, flagellar assembly, lipid biosynthesis/metabolism, heme production, biofilm formation, and two-component regulatory systems. The genomic evidence of heme biosynthesis genes (*hemL*, *hemH*, *hemE*, *hemB*)—encoding glutamate-1-semialdehyde aminotransferase, ferrochelatase, uroporphyrinogen decarboxylase, and porphobilinogen synthase, respectively—corroborates *E. anophelis*’ potential to induce bloodstream infections (Fig. S5).Of particular pathogenic relevance is the bacterium’s biofilm synthesis capability. The ubiquity of biofilm-associated genes (*fleQ*, *fleR*, *flmH*, *ugd*) suggests a critical role for biofilm-mediated colonization in infection dynamics. Classified under the VFDB schema, these determinants span offensive (e.g., invasion), defensive (e.g., immune evasion), nonspecific, and regulatory virulence functions. However, empirical validation remains imperative to conclusively establish the operational role of offensive virulence factors in *E. anophelis* pathogenicity.

## Discussion

*E. anophelis* has risen as a formidable global pathogen, implicated in escalating nosocomial outbreaks, yet remains critically understudied. Understanding its pathogenic mechanisms, antibiotic resistance profiles, and devising targeted interventions are thus imperative. This study bridges critical knowledge gaps by systematically analyzing virulence determinants, resistance genes, and genotype-phenotype correlations in *E. anophelis* isolates, with emphasis on China’s high-burden epidemiological context. Our findings will inform evidence-based strategies to mitigate *E. anophelis*-related morbidity and enhance infection control frameworks.

*E. anophelis* demonstrates intrinsic resistance to most conventional antimicrobial agents [5, 7, 15, 20, 28, 29], though the molecular drivers of this phenotype remain poorly characterized [5, 7, 30, 31]. Our study represents the first comprehensive genotype-phenotype correlation analysis of clinical *E. anophelis* isolates at scale, incorporating coexpression network construction. We establish that genomic resistance determinants only partially account for observed antibiotic resistance profiles. Among all 197 sequenced genomes, three β-lactamase genes (*bla*_BlaB_, *bla*_GOB_, *RASA-1*) were universally present, potentially explaining cephalosporin/carbapenem resistance. This aligns with prior findings: Chang et al. detected metallo-β-lactamases (*bla*_BlaB_, *bla*_GOB−1_) in 37/39 isolates, confirming their role in carbapenem resistance [32], while Lisandro et al. identified BlaB as the primary carbapenemase in *E. meningoseptica* [33]. Notably, our data contradict their observation regarding GOB’s limited contribution to imipenem resistance [33]. Most intriguingly, we observed phenotypic heterogeneity toward penicillins despite conserved β-lactamase genotypes (*bla*_BlaB_, *bla*_GOB_, *bla*_CME_ variants *bla*_CME−1/2/3_), suggesting additional regulatory mechanisms influence resistance expression.

The resistance mechanisms of *E. anophelis* remain poorly characterized, with limited published data available. Emerging evidence demonstrates that *E. anophelis* exhibits intrinsic resistance to multiple antibiotic classes, notably gentamicin, amikacin, tetracyclines (including tigecycline), and trimethoprim-sulfamethoxazole [15, 16, 29, 34, 35]. However, the molecular basis underlying these resistance phenotypes remains largely unexplored in the literature. The observed resistance patterns appear complex and multifactorial. Notably, our findings reveal a paradoxical discordance between predicted resistance genes and actual phenotypic resistance profiles: while most strains lacked identifiable sulfonamide resistance genes, they uniformly displayed resistance to trimethoprim-sulfamethoxazole. Conversely, despite universal carriage of putative tetracycline resistance genes, > 95% of isolates remained susceptible to doxycycline, with all strains sensitive to minocycline. Similarly, although aminoglycoside resistance genes were ubiquitously detected, only 78.2% of clinical isolates demonstrated resistance to amikacin. These findings underscore the critical need for comprehensive investigations into *E. anophelis*-specific resistance mechanisms to facilitate the development of effective therapeutic strategies and inform future antibiotic stewardship efforts.

Mutations in DNA gyrase (GyrA and GyrB) and topoisomerase IV (ParC and ParE) are a key fluoroquinolone resistance mechanism. Lin et al. demonstrated that *E. anophelis* quinolone resistance correlates with *gyrA* mutations (S83I, AGC→ATC; S83R, AGC→AGA), conferring high-level resistance to levofloxacin and ciprofloxacin (MICs > 32 mg/L) [7, 28]. Larkin et al. similarly identified *gyrA* S83I mutations via sequencing but lacked functional validation [36]. Consistently, our study detected multiple *gyrA* mutations (predominantly S83I, S83R, D87N, T409A, N701S, V833L, I834L, A841V, and I842A) in over half of the strains and revealed ubiquitous *gyrB* mutations across all *E. anophelis* isolates. However, despite near-universal mutation carriage, only a minority of strains exhibited corresponding phenotypic resistance. This discrepancy likely reflects limited sample sizes and geographical constraints in prior studies, suggesting *gyrA*/*gyrB* mutations only partially explain fluoroquinolone resistance.

The transmission routes and pathophysiology of *E. anophelis* remain unclear, and no effective preventive measures have been developed to date. Previous studies have suggested potential transmission modes, including vector-borne spread, contaminated aerators and tap water, and vertical transmission [4, 37, 38]. This study’s prediction of virulence-associated factors enhances our understanding of *E. anophelis* pathogenesis and host-pathogen interactions. For instance, the bacterium’s potential to cause bloodstream infections aligns with the presence of heme biosynthesis genes, encoding functional enzymes such as ferrochelatase and porphobilinogen synthase. Bioinformatics analysis revealed a conserved set of putative virulence-associated factors across global *E. anophelis* strains. These factors show partial homology with previously reported virulence genes. Notably, Lin et al. identified several virulence-related genes (e.g., *bplC*, *clpC*, *clpP*, *fleQ*, *galE*, *htpB*, *katA*, *kdtB*, *pilR*, and *sodB*), all of which were also detected in our study’s strains [39]. These genes were similarly present in isolates from the Wisconsin outbreak [40].

In the current study, genomic analysis of *E. anophelis* identified multiple biofilm-associated genes, including *fleQ*, *fleR*, *flmH*, *ugd*, *rmlC*, *capE*, *cpsO*, *capL*, *vipB/tviC*, *uppS*, *motD*, *rmlD*, and *rmlB*, which potentially enhance biofilm formation and reduce antibiotic susceptibility. Notably, the *ugd* gene modulates capsular polysaccharide and liposome synthesis through the PhoP/PhoQ and PmrA/PmrB systems, influencing biofilm development [41]. The regulatory gene *fleQ*, known for its role in *Pseudomonas* biofilm formation, utilizes c-di-GMP signaling pathways to control flagellar biosynthesis and exopolysaccharide production [42, 43]. Intriguingly, recent findings demonstrate that the FleQ-FleN-c-di-GMP complex suppresses the K1-T6SS operon and vgrG1 promoter activity, reducing T6SS expression and antibacterial function [44]. These observations highlight *fleQ’s* critical role in mediating antibiotic resistance mechanisms.

Previous studies have established that outer membrane vesicles (OMVs) and their cargo in Gram-negative bacteria provide valuable insights into bacterial survival strategies, pathogenesis, and antibiotic resistance mechanisms [45–48]. Notably, Zhang et al. demonstrated that upregulated expression of outer membrane modification genes and efflux pump genes enhances β-lactam tolerance in *Acinetobacter baumannii* [49]. These findings suggest that investigating extracellular secretion systems and membrane permeability may reveal novel resistance and virulence mechanisms in *E. anophelis*. Currently, only one comparable study on *E. anophelis* exists. Chiang et al. reported that OMV production plays immunomodulatory roles and is essential for bacterial survival under antibiotic pressure [50]. This underscores the need for further research exploring OMVs in *E. anophelis* pathogenesis and drug resistance.

## Conclusions

In summary, this study demonstrates that *E. anophelis* possesses predominantly intrinsic and intricate resistance and pathogenic mechanisms. Our findings hold substantial implications for clinical antibiotic stewardship. Nevertheless, research on *E. anophelis* drug tolerance and pathogenesis remains in its early stages. Identifying novel molecular targets is essential to develop innovative strategies for preventing and treating *E. anophelis* infections.

## Supplementary Information

Supplementary Material 1: Figure S1. The workflow employed for genomic analysis establishment across 318 global *E. anophelis* isolates. MALDI–TOF MS: matrix-assisted laser desorption ionization time-of-flight mass spectrometry; ANI: average nucleotide identity. Figure S2. Global phylogenetic distribution of antimicrobial resistance genes in *E. anophelis *isolates (expanded version of Fig. 1 with detailed strain information). The left panel shows a maximum-likelihood SNP phylogenetic tree based on 318 isolates. Color-coded columns, ordered from left to right as cluster, origin, region, source, and data, correspond to the legend definitions on the right. The rightmost section specifies the classification and color schemes for each column. Resistance genes are represented by colored blocks (indicating presence) or white blocks (indicating absence), with distinct hues signifying different gene categories: dark green (tetracyclines), brown (sulfonamides), blue (aminoglycosides), lavender (fluoroquinolones), orange-yellow (chloramphenicol), mazarine (beta-lactams), reseda (efflux pump), purple (macrolides), cardinal red (antituberculosis), and yellow (others: EF-Tu, LnuH, ErmF, fusE). Abbreviations: BAL (bronchoalveolar lavage fluid), CSF (cerebrospinal fluid). Figure S3. Sankey diagram illustrating the correlation between strain resistance phenotypes and genotypes. Flow lines depict the distribution of resistant phenotypes and corresponding genotypes. Vertical bars represent individual strains, with line widths proportional to strain abundance (i.e., quantitative distribution ratios). Left nodes (SKLX_A–D) denote four interaction classifications; right nodes indicate antibiotics: PIP (Piperacillin), TZP (Piperacillin-tazobactam), CAZ (Ceftazidime), FEP (Cefepime), IPM (Imipenem), MEM (Meropenem), AZT (Aztreonam), GEN (Gentamicin), AMK (Amikacin), MNO (Minocycline), DOX (Doxycycline), TCY (Tetracycline), TGC (Tigecycline), CIP (Ciprofloxacin), LVX (Levofloxacin), SXT (Trimethoprim–sulfamethoxazole), RFP (Rifampin), VA (Vancomycin), CHL (Chloramphenicol). Figure S4.‌ Antimicrobial resistance profiles of 197* E. anophelis *clinical isolates against 19 tested antibiotics. S: susceptible; I: intermediate; R: resistant. Antibiotic abbreviations are defined in Figure S3. Figure S5. Global phylogenetic distribution of virulence genes across 318 *E. anophelis* isolates (expanded version of Fig. 3 with comprehensive strain annotations). The maximum-likelihood SNP phylogenetic tree and color-coded columnar categories—spanning cluster, origin, region, source, and data—correspond directly to Figure S2. The rightmost segment details column classifications and color schemes consistent with Figure S2. Virulence-associated genes are demarcated by dark green (present) or white (absent) blocks.

Supplementary Material 2.

## Data Availability

The *E. anophelis *bacterial genomes we sequenced have been submitted to the EMBL/GenBank databases and is accessible under project ID PRJNA643387.

## Abbreviations

AMR: Antimicrobial resistance
CARD: Comprehensive Antibiotic Resistance Database
RGI: Resistance Gene Identifier
VFDB: Virulence factor database
SNP: Single nucleotide polymorphism
PGI: Phenotypes-genotypes interaction
T4SS: Type IV secretion system
OMVs: Outer membrane vesicles
