# Supplementary material for: Genomic perspectives on the global dissemination of Elizabethkingia anophelis: unveiling inherent multidrug resistance and virulence determinants
Source: BMC Microbiol. 2026 Feb 27;26:309. doi: 10.1186/s12866-026-04846-7 (PMC13049722; doi:10.1186/s12866-026-04846-7)
Supplement: Supplementary file 1 — Supplementary Material 1: Figure S1. The workflow employed for genomic analysis establishment across 318 global E. anophelis isolates. MALDI–TOF MS: matrix-assisted laser desorption ionization time-of-flight mass spectrometry; ANI: average nucleotide identity. Figure S2. Global phylogenetic distribution of antimicrobial resistance genes in E. anophelis isolates (expanded version of Fig. 1 with detailed strain information). The left panel shows a maximum-likelihood SNP phylogenetic tree based on 318 isolates. Color-coded columns, ordered from left to right as cluster, origin, region, source, and data, correspond to the legend definitions on the right. The rightmost section specifies the classification and color schemes for each column. Resistance genes are represented by colored blocks (indicating presence) or white blocks (indicating absence), with distinct hues signifying different gene categories: dark green (tetracyclines), brown (sulfonamides), blue (aminoglycosides), lavender (fluoroquinolones), orange-yellow (chloramphenicol), mazarine (beta-lactams), reseda (efflux pump), purple (macrolides), cardinal red (antituberculosis), and yellow (others: EF-Tu, LnuH, ErmF, fusE). Abbreviations: BAL (bronchoalveolar lavage fluid), CSF (cerebrospinal fluid). Figure S3. Sankey diagram illustrating the correlation between strain resistance phenotypes and genotypes. Flow lines depict the distribution of resistant phenotypes and corresponding genotypes. Vertical bars represent individual strains, with line widths proportional to strain abundance (i.e., quantitative distribution ratios). Left nodes (SKLX_A–D) denote four interaction classifications; right nodes indicate antibiotics: PIP (Piperacillin), TZP (Piperacillin-tazobactam), CAZ (Ceftazidime), FEP (Cefepime), IPM (Imipenem), MEM (Meropenem), AZT (Aztreonam), GEN (Gentamicin), AMK (Amikacin), MNO (Minocycline), DOX (Doxycycline), TCY (Tetracycline), TGC (Tigecycline), CIP (Ciprofloxacin), LVX (Levofloxacin), SXT (Trime [file 12866_2026_4846_MOESM1_ESM.pdf]

Supplemental Figures

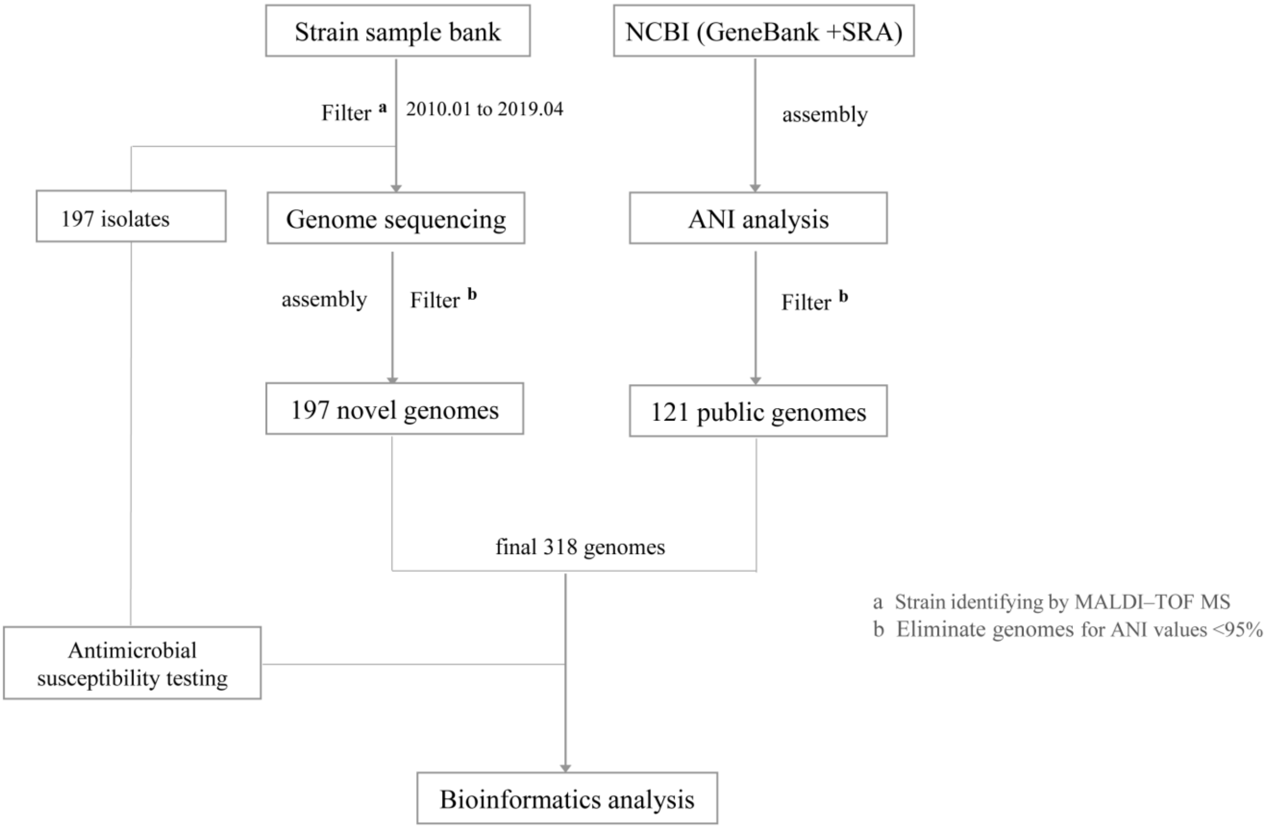

Figure S1. The workflow employed for genomic analysis establishment across 318 global *E. anophelis* isolates. MALDI–TOF MS: matrix-assisted laser desorption ionization time-of-flight mass spectrometry; ANI: average nucleotide identity.

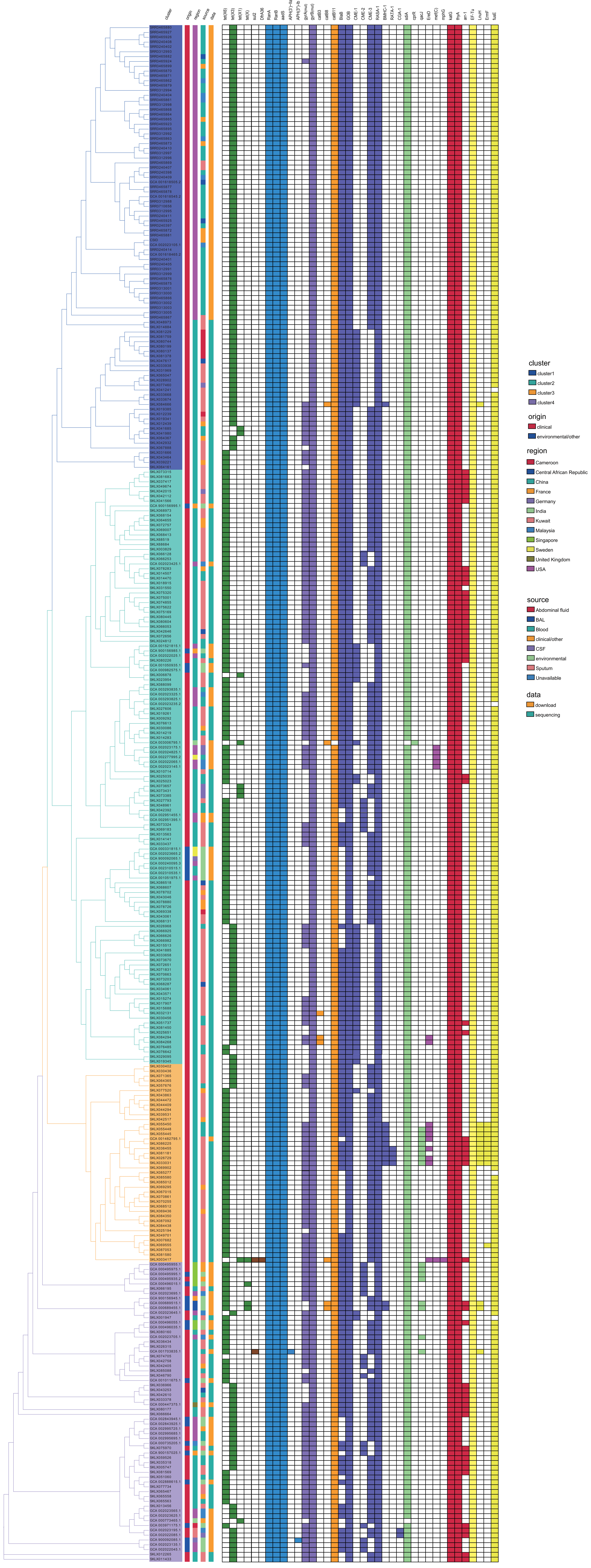
cluster
cluster1
cluster2
cluster3
cluster4
origin
clinical
environmental/other
region
Cameroon
Central African Republic
China
France
Germany
India
Kuwait
Malaysia
Singapore
Sweden
United Kingdom
USA
source
Abdominal fluid
BAL
Blood
clinical/other
CSF
environmental
Sputum
Unavailable
data
download
sequencing

Figure S2. Global phylogenetic distribution of antimicrobial resistance genes in *E. anophelis* isolates (expanded version of Figure 1 with detailed strain information). The left panel shows a maximum-likelihood SNP phylogenetic tree based on 318 isolates. Color-coded columns, ordered from left to right as cluster, origin, region, source, and data, correspond to the legend definitions on the right. The rightmost section specifies the classification and color schemes for each column. Resistance genes are represented by colored blocks (indicating presence) or white blocks (indicating absence), with distinct hues signifying different gene categories: dark green (tetracyclines), brown (sulfonamides), blue (aminoglycosides), lavender (fluoroquinolones), orange-yellow (chloramphenicol), mazarine (beta-lactams), reseda (efflux pump), purple (macrolides), cardinal red (antituberculosis), and yellow (others: EF-Tu, LnuH, ErmF, fusE). Abbreviations: BAL (bronchoalveolar lavage fluid), CSF (cerebrospinal fluid), gyrA(mut): gyrA mutants, gyrB(mut): gyrB mutants.

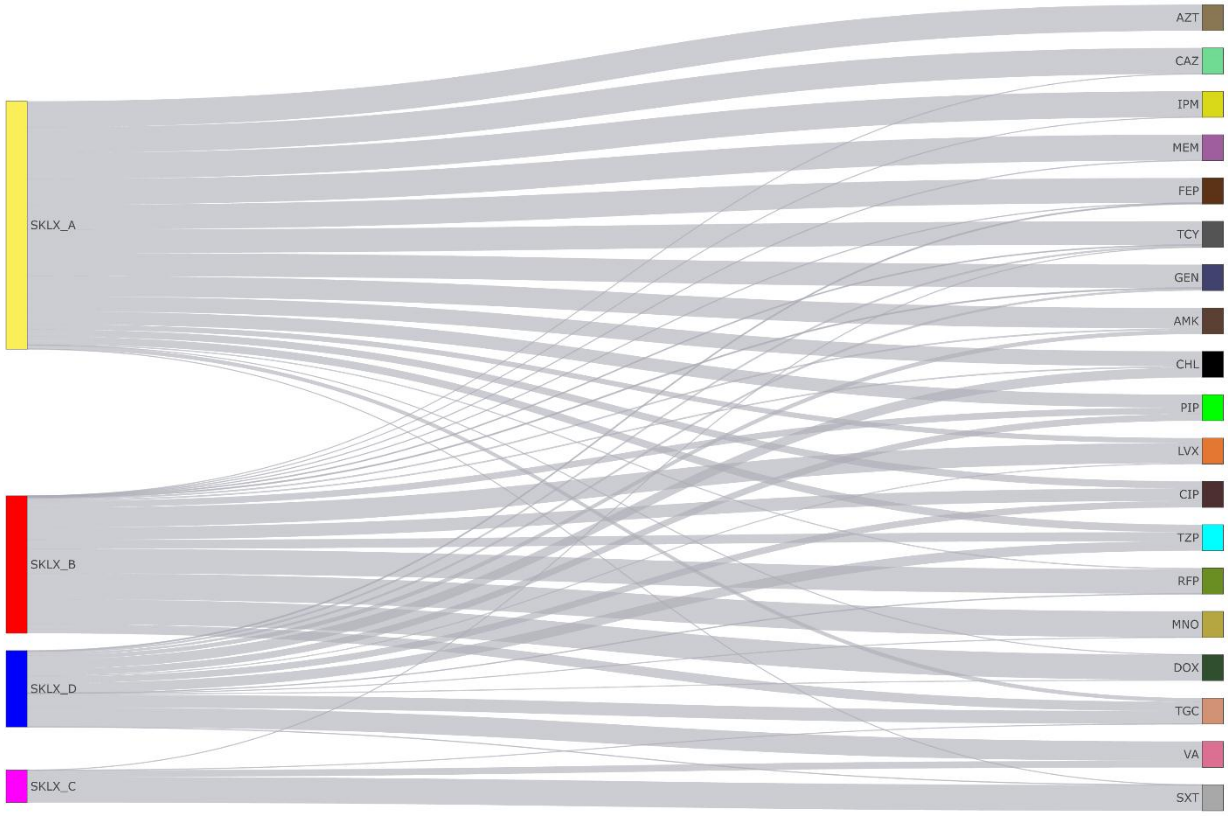

Figure S3. Sankey diagram illustrating the correlation between strain resistance phenotypes and genotypes. Flow lines depict the distribution of resistant phenotypes and corresponding genotypes. Vertical bars represent individual strains, with line widths proportional to strain abundance (i.e., quantitative distribution ratios). Left nodes (SKLX_A–D) denote four interaction classifications; right nodes indicate antibiotics: PIP (Piperacillin), TZP (Piperacillin-tazobactam), CAZ (Ceftazidime), FEP (Cefepime), IPM (Imipenem), MEM (Meropenem), AZT (Aztreonam), GEN (Gentamicin), AMK (Amikacin), MNO (Minocycline), DOX (Doxycycline), TCY (Tetracycline), TGC (Tigecycline), CIP (Ciprofloxacin), LVX (Levofloxacin), SXT (Trimethoprim–sulfamethoxazole), RFP (Rifampin), VA (Vancomycin), CHL (Chloramphenicol).

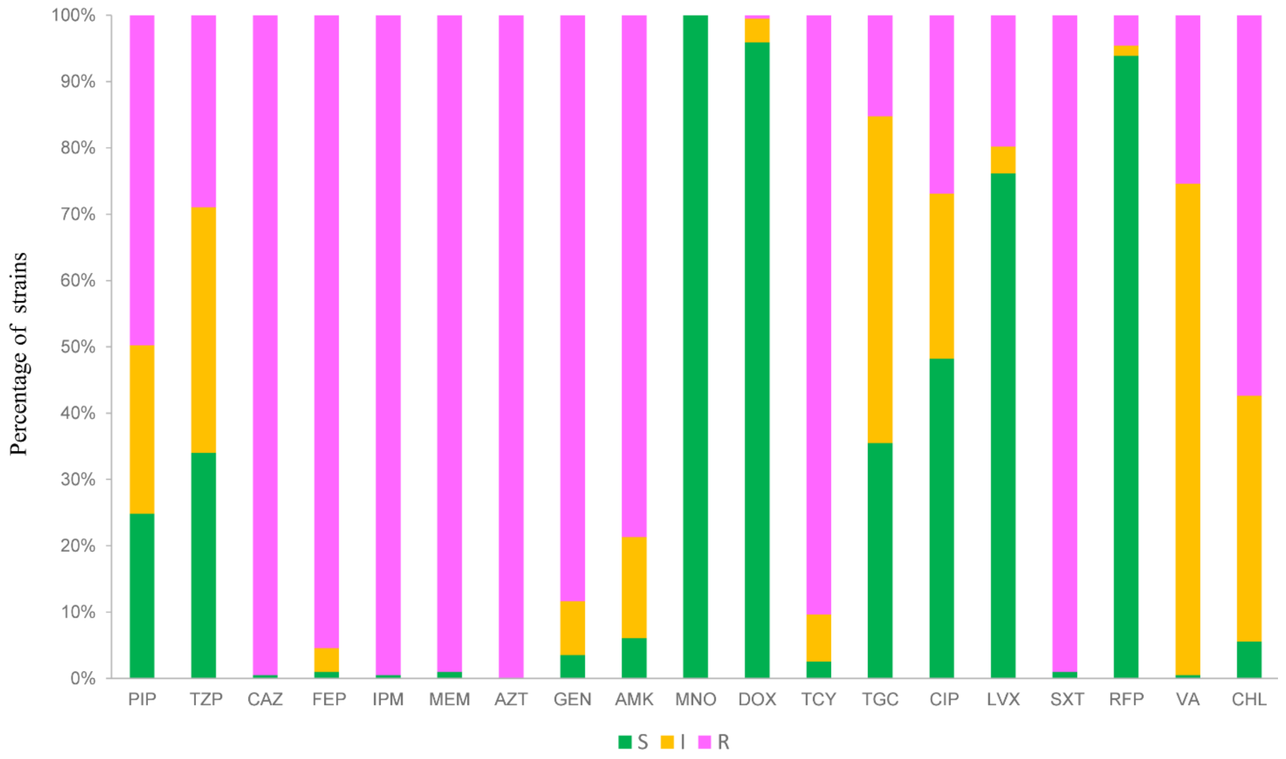

Figure S4. Antimicrobial resistance profiles of 197 *E. anophelis* clinical isolates against 19 tested antibiotics. S: susceptible; I: intermediate; R: resistant. Antibiotic abbreviations are defined in Figure S3.

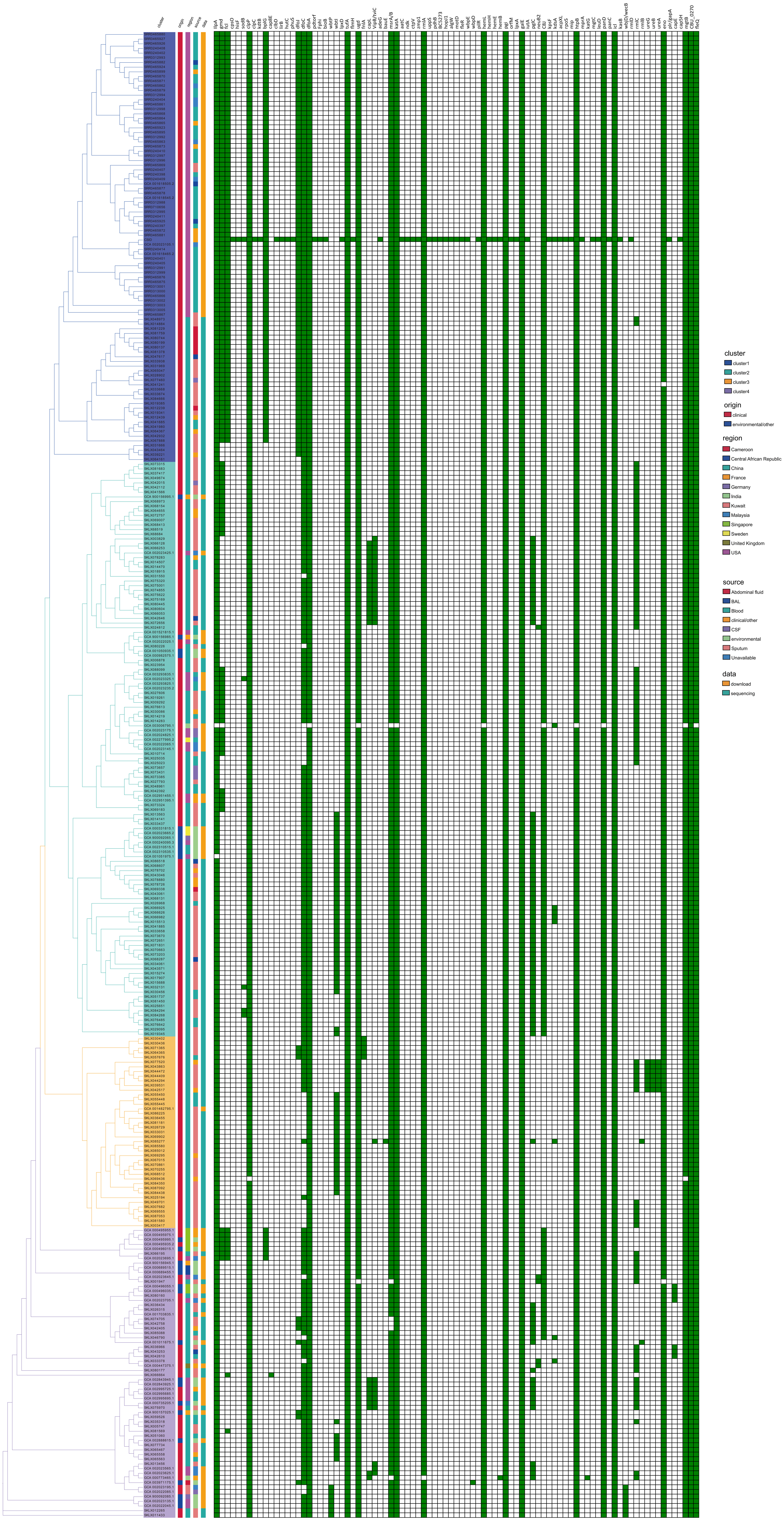

cluster
origin
region
source
data
llpA
gmd
fcl
cpsO
hisF
sodB
clpP
clpC
kdtB
bplG
cap8E
clbD
lirB
hutC
phuS
dfoJ
dfoC
dfoA
pdtorf
Fphi
bioB
wbtP
wbtI
lpxD
tufA
flmH
ugd
fnlA
capL
VipB/tviC
adeG
bauE
msrA/B
katA
aatC
ndk
ctpV
zmp1
rmlA
uppS
pdhB
BC5273
hopJ1
algW
motD
fleR
wbpE
wbpD
pilR
hemL
hemH
hemE
hemB
pgi
orfM
lpxA
galE
iutA
pglC
neuB2
C8J
kpsF
kdsA
acpXL
rpoS
mip
htpB
mprA
katG
mgtC
leuD
panD
panC
icl
kasB
wbjD/wecB
rmlD
rmlC
rmlB
ureG
ureB
ureA
eno
plr/gapA
capE
cap5H
mgtB
CBU_0270
fleQ
cluster
cluster1
cluster2
cluster3
cluster4
origin
clinical
environmental/other
region
Cameroon
Central African Republic
China
France
Germany
India
Kuwait
Malaysia
Singapore
Sweden
United Kingdom
USA
source
Abdominal fluid
BAL
Blood
clinical/other
CSF
environmental
Sputum
Unavailable
data
download
sequencing

Figure S5. Global phylogenetic distribution of virulence genes across 318 *E. anophelis* isolates (expanded version of Figure 3 with comprehensive strain annotations). The maximum-likelihood SNP phylogenetic tree and color-coded columnar categories—spanning cluster, origin, region, source, and data—correspond directly to Figure S2. The rightmost segment details column classifications and color schemes consistent with Figure S2. Virulence-associated genes are demarcated by dark green (present) or white (absent) blocks.
